# Circumscription and lectotypification of *Hedychium villosum* and its variety *H. villosum* var. *tenuiflorum* (Zingiberaceae)

**DOI:** 10.3897/phytokeys.25.4113

**Published:** 2013-08-21

**Authors:** Edakkandiyil Sanoj, Mamiyil Sabu, Ayillath Kuttiyil Pradeep

**Affiliations:** 1 The Zamorin’s Guruvayurappan College, G.A. College P.O., Kerala-673014, India; 2 Department of Botany, University of Calicut, Kerala-673635, India

**Keywords:** Gingers, India, nomenclature, typification, Nathaniel Wallich, Zingiberaceae

## Abstract

The nomenclatural confusion between the Indian gingers *Hedychium villosum* Wallich and its variety *Hedychium villosum* var. *tenuiflorum* (Wall. ex Voigt) Wall. ex Baker is discussed. Both taxa are lectotypified in order to stabilize the names and contribute towards a resolution of their confusing nomenclatural past. Both taxa are described in detail to aid identification.

## Introduction

Zingiberaceae are one among the ten largest monocotyledonous families in India, represented by 20 genera and about 200 species, and are mainly concentrated in the Northeastern, Peninsular and Andaman and Nicobar regions. *Hedychium*, commonly called “ginger lily” or “butterfly lily” produces one of the most beautiful and aromatic flowers in the family Zingiberaceae. The genus *Hedychium* was established by Koenig (1783) with *Hedychium coronarium* J.Koenig as the only species, based on Rumphius’s (1747) illustration. *Hedychium* includes about 80 species with highest species diversity in the eastern Himalaya region to South China and Southeast Asia. About half the species occur in the Indochinese region (Sirirugsa and Larsen 1995). *Hedychium* is the largest genus of Zingiberaceae in India, with about 43 taxa (Jain and Prakash 1995), mostly restricted to Northeastern India.

**Figure 1.**
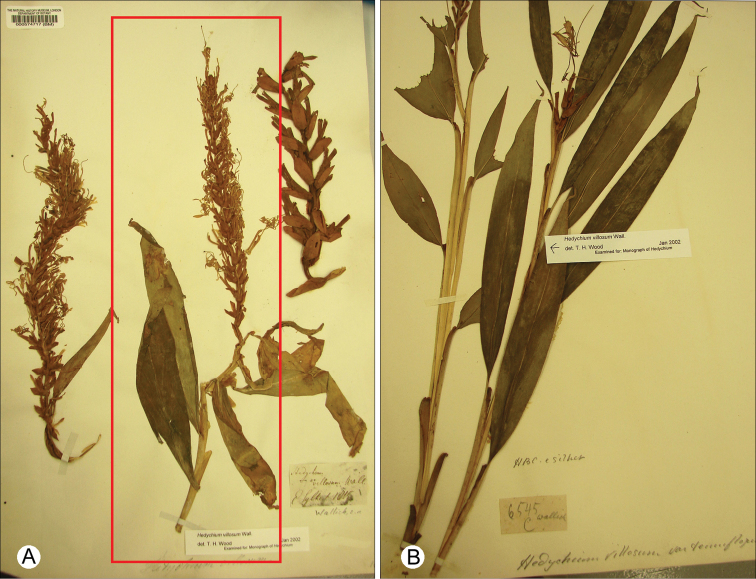
Type specimens: **A** *Hedychium villosum* Wall. - Sylhet, 1815, *M. R. Smith s.n. pro parte* (middle specimen; BM000574696) **B** *Hedychium villosum* var. *tenuiflorum* (Wall. ex Voigt) Wall. ex Baker- Botanic Garden Calcutta, originally from Sylhet, *N. Wallich 6545C* (BM).

Whilst revising the species of *Hedychium* in India, nomenclatural and circumscription problems were encountered in relation to two plant names coined by Nathaniel Wallich, *Hedychium villosum* Wall. and its variety, *Hedychium villosum* var. *tenuiflorum* (Wall. ex Voigt) Wall. ex Baker. The plants of these two taxa are very peculiar in their sagittate anthers and winter flowering (December to April), whereas in other Indian species of *Hedychium* the anthers are oblong and plants flower during the monsoon season (June through October). For a long time, *Hedychium villosum* was identified as *Hedychium villosum* var. *tenuiflorum* or vice-versa or the two taxa were even lumped as a single highly variable species. As a result of extensive studies of the literature, herbarium and live specimens, we are now able to identify and delimit these two taxa, and recognize them as distinct. The variety differs in its shorter inflorescence, larger white flowers and larger anthers (see Table 1, Figs 1A, B). Confusion over taxon identity was in part caused by poor knowledge of the types of these names.

**Table 1.** Main morphological differences between *Hedychium villosum* and *Hedychium villosum* var. *tenuiflorum*.

**Characters**	***Hedychium villosum***	***Hedychium villosum* var. *tenuiflorum***
Inflorescence	16.5–33 cm long	8.4–12.3 cm long
Flowers	4.7–5.2 cm long, pale yellow	11.8–12.2 cm long, white
*Calyx*	1.6–1.8 cm long	3.3–3.7 cm long
Corolla tube	1.9–2.1 cm long, densely pubescent externally	5–5.2 cm long, glabrous externally
Corolla lobes	1.5–1.7 cm long, lower half pubescent externally	4–4.4 cm long, glabrous externally
Lateral staminodes	1.3–1.5 × c. 0.15 cm	3.3–3.5 × 0.1–0.15 cm
Labellum	1.3–1.4 × c. 0.5 cm	3.1–3.5 × 1.2–1.5 cm
Filament	2.4–2.6 cm long	5.3–6.3 cm
Anther	c. 1.5 × 1.5 mm	c. 3.5 × 2.5–3 mm

## Nomenclatural discussion

*Hedychium villosum* was originally described by Wallich (in Roxburgh 1820) from a specimen sent to him by Mathew Robert Smith from the “mountains north-east of Bengal”. He described it as a native of northeast of Bengal, flowers in rainy season and “*Kattia Ram Rait*” as its local name (Khasee language). In describing the species he compared it with a live specimen of *Hedychium gracilie* Roxb. and differentiated in its larger plant size, length and villosity of inflorescence, copious fascicled flowers, and corolla lobes of equal length. From Wallich’s description, it is evident that this is a species with a cylindrical inflorescence of 25–30.5 cm long, with small, pale yellow flowers (corolla tube c. 3.8 cm long, bracts much shorter than tube, calyx somewhat shorter than tube, filament as long as tube and scarlet). At Kew we located a colour plate drawn for Wallich that closely resembles the description of *Hedychium villosum* Wall. Wallich also incidentally mentioned “var. *tenuiflorum*” under this taxon in his catalogue (Numer. List. 1832) for Wallich 6545C, but did not formally publish this name nor include it in his later publications under *Hedychium villosum* (Wallich 1853, 1855). Indeed, it is evident from Wallich’s own statement (“they differ not even as varieties I believe”), that he preferred not to recognize his “var. *tenuiflorum*” as a distinct taxon. Wallich (1832) cited it as “6545-C: *Hedychium villosum* Wall. ? var. *tenuiflorum* Wall. HBC” (Hortus Botanicus Calcutta) “*e Sillet*” (from Sylhet).

A few years later, Roscoe (1827) while treating ‘Monandrian Plants of the order Scitamineae’, accepted Wallich’s broad view and did not recognise any infraspecific taxa under *Hedychium villosum*. It is evident from his observation, “the chief diversity we have observed between our plant and that described in the ‘Flora Indica’, is in the downy margin and mid-rib of the leaves, and in the colour of the flowers, which in the ‘Flora Indica’ are described as of a pale yellow colour, whilst in ours they are a pure white”, that he had not recognized any taxa at intraspecific level probably for the reason that the sagittate anther peculiar to *Hedychium villosum* is also present in the other element (Wallich’s “*tenuiflorum*”). He stated further that, “Dr. Wallich has also communicated another species under the name *Hedychium tenuiflorum*, which resembles the present, as well in the deep-lobed, undulated lip, as in the small sagittateanther.....”. Interestingly, the description and figure provided by Roscoe (1827, t. 54) under the name *Hedychium villosum* are an excellent match for ‘var. *tenuiflorum*’ (*sensu* the specimen Wallich 6545C), not for *Hedychium villosum* (*sensu* Roxburgh 1820).

Voigt (1845) recognised *Hedychium villosum* in the strict sense of Wallich (in Roxburgh 1820) by specifically excluding Roscoe’s concept (“*Hedychium villosum* Wall. not Roscoe”), and by including *Hedychium tenuiflorum* Wall. as *Hedychium villosum* of Roscoe and not of Wallich (“*Hedychium tenuiflorum* Wall. -*Hedychium villosum* Roscoe, not Wall.”). Our study of a large number of both live and herbarium specimens, including consultation of types, protologues and other relevant literature corroborates this view (Voigt 1845). We feel that these two taxa are distinct but that they only warrant recognition at varietal, rather than specific rank. Hence, we recognize two taxa: *Hedychium villosum* var. *villosum* as the nominal species and a distinct variety, *Hedychium villosum* var. *tenuiflorum.*

During herbarium studies of Wallich material we were unable to locate material identifiable as *Hedychium villosum* in the original Wallich herbarium at Kew. However, at BM we located one specimen collected from Sylhet in 1815 that corresponded to that cited by Roxburgh and Wallich. The label on this specimen is in Wallich’s hand. Although the specimen lacks a collector name, we infer from the protologue that it was collected by M.R. Smith. It is mounted with another two plant fragments, the right hand material corresponds to *Hedychium villosum* var. *tenuiflorum* and other the central and left hand specimens match what is currently understood as *Hedychium villosum*. Hence, from the elements on this sheet, the middle plant fragment is selected and designated here as the lectotype of *Hedychium villosum*. Because Voigt (1845) referred to Wallich through reference to Roscoe in his description of the species *Hedychium tenuiflorum*, he implicitly included the specimen *Wallich 6545C*. We did not locate any material unambiguously referable to this collection and locality in the Wallich herbarium at Kew, but we did locate a specimen of *Wallich 6545C* at BM collected from the Botanic Garden Calcutta, originally from Sylhet; we designate this specimen here as the lectotype of the *Hedychium villosum* var. *tenuiflorum.*

## Taxonomic treatment

### Hedychium villosum villosum

Fl. Ind. (ed. Carey) 1: 12. 1820.

#### Type.

**Figure 2.**
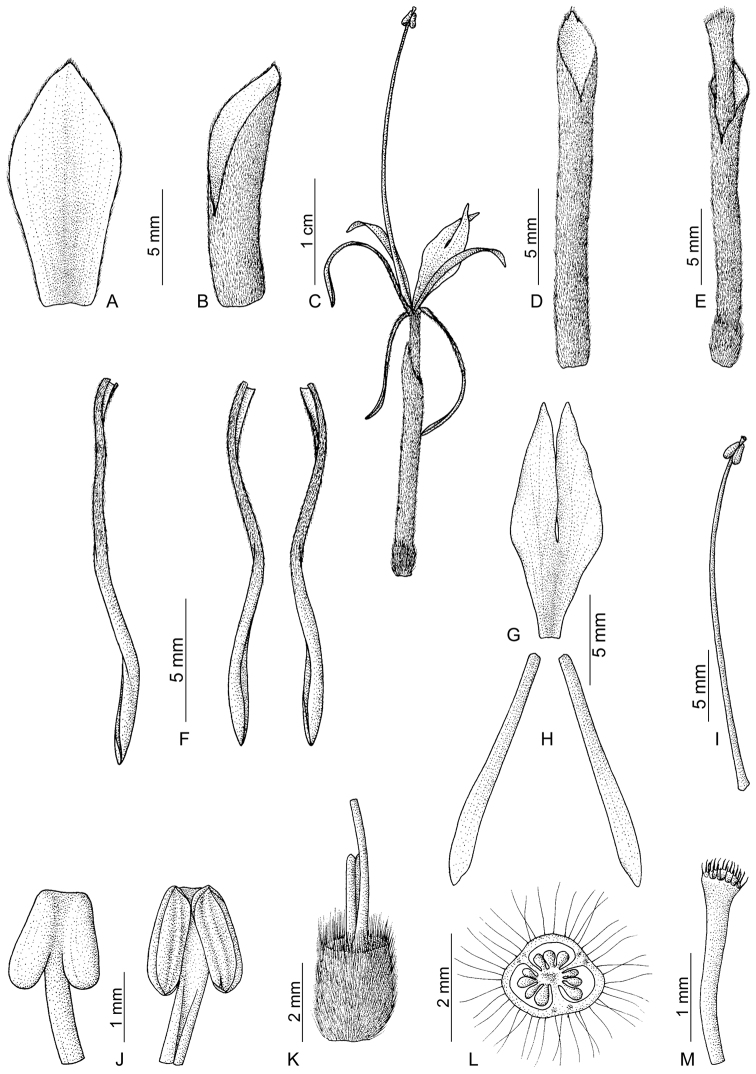
*Hedychium villosum* Wall. **A** bract **B** bracteole **C** single flower **D** calyx **E** corolla tube with calyx and ovary **F** corolla lobes **G** labellum **H** lateral staminodes **I** stamen **J** anther, back view (left) and front view (right) **K** ovary with epigynous glands and base of style **L** cross section of ovary **M** stigma with a part of style. Line drawing by E. Sanoj; voucher *M. Bhaumik 1922* (CAL).

India. “Sylhet”, 1815, *M. R. Smith* *s.n*. *pro parte* (middle specimen) (lectotype, designated here: BM! [BM000574717, middle stem only]). Figs 1A, 2.

#### Description.

Epiphytic perennial herbs. *Leafy shoots* 60–90 cm high, erect, slender. *Leaves* 8–12 in number, spreading, at a distance of 3.4–7.5 cm, sessile; sheath 1.9–2 cm wide, puberulent at margins; ligule 1.8–2.1 × 0.7–0.8 cm, single-lobed, oblong, membranous, puberulent externally, tip acute; lamina 14–20 × 3.7–4.5 cm, elliptic-lanceolate, dark green and glabrous above, pale green and puberulent below; midrib pubescent below; tip long acuminate; base obtuse; margin non-ciliate, translucent, white tinged. *Inflorescence* 16.5–33 cm long, erect or drooping, lax, cylindrical. *Bract* 1.2–1.4 × 0.4–0.5 cm, one on each flower, convolute, elliptic, boat-shaped, brown, non-tubular, more or less leathery, densely hairy externally, hairs brown; margin non-ciliate; cincinnus 2–3 -flowered. *Bracteoles* 1–1.3 × c. 0.25 cm, brown, tubular, unilaterally split upto 5–8 mm, densely hairy externally, hairs brown; margin non-ciliate. Flower 4.7–5.2 cm long, pale yellow, highly fragrant, many open at a time. *Calyx* 1.6–1.8 × c. 0.15 cm, tubular, unilaterally split up to 5–6 mm, gradually convolute towards the tip, membranous, translucent, densely pubescent externally; hairs brown; obscurely 3 -lobed at tip. *Corolla tube* 1.9–2.1 cm long, c. 1.5 mm wide at mouth, straight, exceeding the calyx and bract, pale yellow, densely pubescent externally, hairs brown, hairy internally. *Corolla lobes* oblong, pale yellow, lower half pubescent externally, glabrous on upper half, margins non-ciliate, non-beaked at tip, lobes 3-nerved; dorsal lobe 1.6–1.7 × c. 0.15–0.2 cm long; lateral lobes 1.5–1.6 × c. 0.15 cm long. *Lateral staminodes* 1.3–1.5 × c. 0.15 cm, linear, straight, pale yellow, tip acute. *Labellum* 1.3–1.4 × c. 0.5 cm, elliptic, narrow towards base, pale yellow, sinus 0.8–0.9 cm deep, lobes tip acute; claw c. 1 mm wide at base. *Stamen* 2.6–2.8 cm long. *Filament* 2.4–2.6 cm long, scarlet, straight, c. 0.5 mm wide at base. *Anther* c. 1.5 × 1.5 mm, sagittate, brown, glabrous, non-crested, pink, truncate at tip, glabrous. *Ovary* c. 3 × 2 mm, oblong, pale green, densely pubescent externally. *Style* filiform, white, glabrous. *Stigma* cup-shaped, margin ciliate, c. 0.5 mm exserted from anther. *Epigynous glands* two, 2.5–3 mm long, oblong. *Fruits* unknown.

#### Distribution.

Bangladesh, China, India and Myanmar from 660 to 1400 m (Wu and Larsen 2000).

#### Indian specimens examined.

**INDIA. Arunachal Pradesh:** Changlang Dt.: Chenglang to Khela, 666.6 m, 12 Mar 1958, *G.K. Murthy 12939* (ASSAM); Dibang Valley Dt.: Thewarygaon, 1200 m, 20 May 1998, *M. Bhaumik 1922* (CAL); Anini, 1640 m, *M. Bhaumik* *s.n.* (CAL); Mehao Lake, 1300 m, 1 Dec 1996, *M. Bhaumik & M.K. Pathak 1153* (CAL); 1200 m, 20 May 1998, *M. Bhaumik & M.K. Pathak 1902* (CAL); West Siang Dt.: Nokka to Nagminu, 5 Jul 1961, *D.B. Deb 26547* (ASSAM); **Meghalaya:** Khasia, 4000 ft, *J.D. Hooker & J. Thomson s.n.* (K), Khasia No. 6 (CAL); **Mizoram:** Aizwal Dt.: Sialsuk, Lushai Hills, 4700 ft, 15 Jan 1963, *D.B. Deb 30713* (ASSAM); Lunglei Dt.: Theiriat, Lushai Hills, 25 Jan 1963, *D.B. Deb 31265* (ASSAM); Serchip Dt.: Kheitum, Lushai Hills, 23 Jan 1963, *D.B. Deb 31185* (ASSAM); **Locality unknown.** *Mack* *s.n.* (K), *Griffith* *s.n*.

### Hedychium villosum tenuiflorum

(Wall. ex Voigt) Wall. ex Baker, Fl. Brit. India [Hooker] 6: 229. 1892 (excl. description).

#### Type.

**Figure 3.**
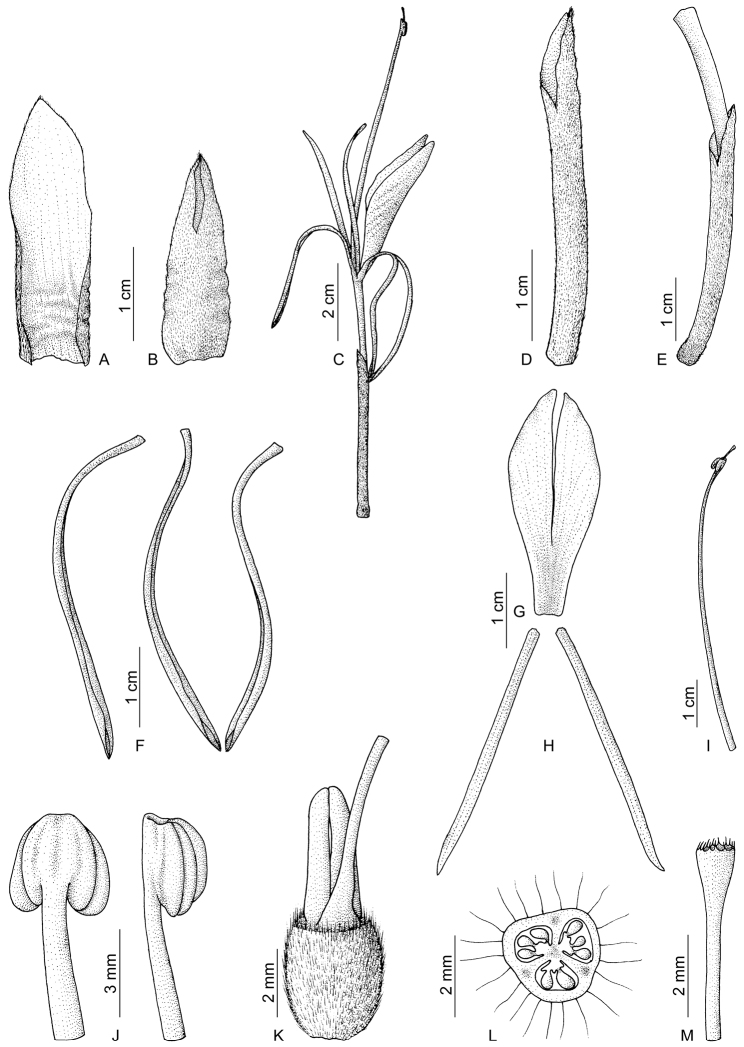
*Hedychium villosum* var. *tenuiflorum* (Wall. ex Voigt) Wall. ex Baker. **A** bract **B** bracteole **C** single flower **D** calyx **E** corolla tube with calyx and ovary **F** dorsal and lateral corolla lobes **G** labellum **H** lateral staminodes **I** stamen **J** anther, back view (left) and lateral view (right) **K** ovary with epigynous glands and base of style **L** cross section of ovary **M** stigma with a part of style. Line drawing by E. Sanoj; voucher *E. Sanoj 95619* (CALI).

India. Botanic Garden of Calcutta, originally from Sylhet, *N. Wallich 6545C* (lectotype, designated here: BM!) Figs 1B, 3.

#### Description.

Epiphytic perennial herbs. *Leafy shoots* 66–70 cm high, slanting or erect. *Rhizomes* 1.7–3 cm wide, pale green internally, slightly aromatic. *Roots* 0.6–12 mm wide, velamen type. *Leaves* 5–10 in number, at a distance of 4–8.5 cm, sessile; sheath 1.9–2 cm wide, green, margins pink, pubescent externally; ligule 2.9–3.4 × 1.3–1.6 cm, single-lobed, oblong, pale pink, densely pubescent externally, tip acute, margins and tip brown, early dried off; lamina 34–40 × 8–9 cm, elliptic-lanceolate, dark green above, pale green below, glabrous; midrib pubescent below, becomes glabrous towards tip; tip long acuminate; base obtuse; margin non-ciliate, translucent, pale pink tinged. *Inflorescence* 8.4–12.3 cm long, erect, lax, cylindrical. *Bract* 2.7–2.9 × 1.1–1.2 cm, one on each flower, convolute, lanceolate, boat-shaped, brown, non-tubular, leathery, easily dried before flowering, densely hairy or villose externally, hairs brown; margin ciliate towards tip; cincinnus 3–4 -flowered. *Bracteoles* 2.1–2.2 × 0.7–0.75 cm, lanceolate, brown, tubular, unilaterally split upto 9–10 mm, somewhat leathery, densely hairy or villose externally, hairs brown; margin ciliate towards time. *Flower* 11.8–12.2 cm long, white with red stamen, mildly fragrant, three-many opens at a time. *Calyx* 3.3–3.7 × 0.2–0.3 cm, pale green, pink tinged towards tip, tubular, unilaterally split up to 5–9 mm, lower 2/3 portion closely appressed to corolla tube, membranous, translucent, densely pubescent externally; tip easily dried while flowering, obscurely 2 or 3 -lobed. *Corolla tube* 5–5.2 cm long, 2–2.5 mm wide at mouth, straight, exceeding the calyx and bract, creamy white, translucent towards base, glabrous externally, hairy internally. *Corolla lobes* oblong, greenish white, glabrous, drooping from flower; tip non-pouched, margins non-ciliate, lobes 3-nerved; dorsal lobe 4.3–4.4 cm long; lateral lobes 4–4.1 cm long. *Lateral staminodes* 3.3–3.5 × 0.1–0.15 cm, linear, straight, linear, white with a slight yellow tinge at base; tip acute, rarely forked. *Labellum* 3.1–3.5 × 1.2–1.5 cm, oblanceolate, boat-shaped, white with a yellow tinge at base, sinus 1.9–2.2 cm deep, lobes ensiform, unequal; claw 4–4.5 mm wide at base. *Stamen* 5.8–6.5 cm long. *Filament* 5.3–6.3 cm long, straight, c. 1.5 mm wide at base, red, light red towards tip. *Anther* c. 3.5 × 2.5–3 mm, sagittate, brown, glandular hairy; connective red, glabrous, row of hairy at margin, tip prolonged in to a crest; crest c. 1 mm long, red, truncate or slightly notched at center, glabrous. *Ovary* 2.5–3 × 2–2.5 mm, barrel-shaped, pale green, densely pubescent externally. *Style* filiform, white, glabrous, pale pink spotted towards stigma. Stigma cup-shaped, margin ciliate, 2–5 mm exserted from anther. *Epigynous glands* two, 3.5–5.5 mm long, oblong-lanceolate, yellow, free at base, fused towards tip. *Fruits* 1–1.1 × 1–1.1 cm, globular, sericeous, slightly angular.

#### Distribution.

Northeastern India, Bangladesh, northern Myanmar, southern Yunnan and Guangxi provinces of China, Thailand, Vietnam, and Malaysia from 600 to 1800 m (Wu and Larsen 2000).

#### Indian specimens examined.

**INDIA**. **Arunachal Pradesh**: Changlang Dt.: Wakka to Nagminu, 5 Jul 1961, *D.B. Deb 26547* (ASSAM, CAL); Chenglang to Khela, 666.6 m, 12 Mar 1958, *G.K. Murthy 12939* (ASSAM, CAL); Dibang Valley Dt.: Delei Valley, 28°21'N, 96°37'E, 14 Jul 1928, *F.K. Ward 8448* (K); EAST SIANG Dt.: Rengging, Flora of Abor expedition, 24 Feb 1912, *J.H. Burkill 36708* (CAL); Lohit Dt.: Dreyi above Denning, 28°10'N, 96°15'E, 2000–4000 ft, 5 Mar 1928, *F.K. Ward 7916* (K); Forest around Hayuliang along Dalai River, 13 Jan 1970, *J. Joseph 48932* (ASSAM); **Assam**: Kamrup Dt.: Golaghat Dt.: Jabocka, Naga Hills, 6000 ft, Mar 1899, *Prain’s Collector 99* (CAL); Lakhimpur Dt.: Kakopathar, 23 Feb 1947, *M.M. Srinivasan 21955* (ASSAM); **Manipur**: s.loc., 22 Jan 1953, *D. Deb 682* (CAL); Koupia N.W. range, 4-6000 ft, 10 Feb 1882, *G. Watt 5844* (CAL, E, K); Imphal Dt.: Kauglatonghi, *s.coll. 10815* (K), 3000 ft, Feb 1906, *A. Meebold 10815* (CAL), 3500 ft, 5 Mar 1946, *J. Hake 980* (K); **Meghalaya**: Khasia, 4000 ft, *J.D. Hooker & J. Thomson s.n.* (K), s.loc. 1845, *Griffith 45* (K), s.loc. 5000 ft, 1878, *Geo*. *Gallatly 108* (CAL), *Native collectors of Bot. Garden Calcutta* *s.n.* (E), 5–6000 ft, Feb 1906, *A. Meebold 5179* (CAL), 8 Mar 1921, *U. Kanjilal 7660* (ASSAM); East Khasi Hills Dt.: Baedon Falls, Shillong, 22 Mar 1892, *D. Prain 43* (CAL); Pynursla forest, 18 Oct 1938, *R.N. De & D.C. Forests 19615* (ASSAM); Mawrynklang, near Wattle Plantation, 27 Jan 1957, *G.K. Deka 5232* (ASSAM, CAL); Mawswai, K&J Hills, 1200 m, 26 Mar 1960, *G. Panigrahi 21338* (ASSAM); Woodlands, 11 Mar 1966, *D.M. Verma 36862* (ASSAM); Barapani, 5000 ft, Geo Gallatly 108 (K); Jorain, 1580 m, 13 Aug 2004, *E. Sanoj 95619* (CALI); Jaintia Hills Dt.: Lumsnong, 4 Jan 1958, *A. Sanyal 9* (CAL); West Khasi Hills Dt.: Nongstoin village, 9 Mar 1972, *P.C. Pant 51483* (ASSAM); **Mizoram**: Zopui-Tlang, 1332 m, 22°50'83.2"N, 092°48'80.5"E, 07 Sep 2002, *M.G. Prasanth Kumar & Jana Leong-Škorničková 86213* (CALI); Lunglei Dt.: Near Fort Lungleh, 3-4000 ft, Apr 1899, *A.T. Gage 120* (CAL); Lushai Hills, Darzo, 5000 ft, Jan 1928, *N.E. Parry 581* (K); **Nagaland**: Naga Hills, Pipluma, 1 Mar 1882, *s.coll. 158* (CAL), Naga hills, Jun 1935, *N.L. Bor 21217* (ASSAM), Jisi, 5000 ft, 6 Mar 1955, *N.L. Bor 2871* (K); Kohima Dt.: Tseminyu forest-Wokha Road, 12 Apr 1975, *Chandra Bahadur 61794* (ASSAM); Tuensang Dt.: 5 Km from Noklak, 20 May 2007, *V.P. Thomas & V.A. Muhammed Nissar 103700* (CALI); **Locality unknown**. *Mack* *s.n.* (K); *C.B. Clarke* *s.n.* (K); *Hooker & Thomson 6545* (K); *N.E. Parry* *s.n.* (CAL); *Griffith* *s.n.* (CAL).

##### Nomenclatural confusion surrounding these names

In 1890, probably following Roscoe (1827), C.B. Clarke annotated two herbarium sheets at Kew, each of which is a single sheet with two specimens mounted on it. He determined the left hand specimens of both sheets (K000640488! and K000640489!) bearing long cylindrical inflorescences and rather small flowers and anthers as ‘var. *tenuiflorum* Wall.’, and the right hand specimens (K000640486! and K000640487!) bearing rather large flowers and anthers as *Hedychium villosum*. It appears that subsequent authors (Baker 1892; Schumann 1904) treated *Hedychium villosum* Wall. not as understood by Wallich (in Roxburgh 1820) but in the sense of Roscoe (1827) and Clarke’s annotation of the Kew sheets in 1890. Further propagating this error, Baker (1892) attempted to provide a short description for Wallich’s variety, var. *tenuiflorum*, for the first time and cited original Wallich material - *Wallich 6545C* (he incorrectly cited “6546 C”, a correctable error). Unfortunately his description (“flowers much smaller than the type, corolla-segments staminodes and lip ½ in”) is not applicable to var. *tenuiflorum* (*sensu Wallich 6545C*), but to the typical variety. Baker cited Roscoe’s plate (Monandr. Pl. Scitam. t. 54. 1827) and considered the white and large-flowered plant as the typical *Hedychium villosum* Wall., not the pale yellow and small flowered one as Wallich (in Roxburgh 1820) originally described the species.

Schumann (1904) recognized *Hedychium villosum* var. *tenuiflorum* as a distinct species under the name *Hedychium tenuiflorum* (Baker) K. Schum. He differentiated his distinct species by the nature of inflorescence, less than 1 mm long anthers, narrower leaves and smaller flowers. As with Baker (1892), Schumann’s description of the taxon (except “*corolla prob. albae*”) -“*Folia omnia summa ipsa sessilia stricte lanceolata longissime attenuato-acuminata et rostrata acutissima basi angustata utrinque glabra... .., Spica 25-30 cm longa anguste cylindrical... .., anther vix 1 mm longa*” perfectly agrees with what is currently understood as *Hedychium villosum* Wall. Inspection of specimens cited, such as *Hooker & Thomson s.n.* (K!), *Griffith 5661* (K!) and *Prain 43* (CAL!) supports our contention that this is what Schumann did. Schumann (1904) also cited a specimen from Silhet, *Wallich 6545A* both for his *Hedychium tenuiflorum* and *Hedychium villosum*. Although he mentioned Robert Smith’s introduced material in 1815 under *Hedychium villosum*, his description does not agree perfectly with Wallich’s protologue. There are two sheets in the microfiche of the Wallich Catalogue bearing catalogue number 6545A. Of the two sheets labelled as Wall. Cat. n. 6545A, the first sheet contains two specimens of *Hedychium villosum* var. *tenuiflorum* and second one is a composite of two taxa, the top left material corresponding to *Hedychium villosum* and the other two specimens matching what we currently recognize as *Hedychium villosum* var. *tenuiflorum*. Judging from the description and materials cited by Schumann (1904), it appears that he treated *Hedychium villosum* in the sense of Roscoe (1827), not in the sense of Wallich.

## Supplementary Material

XML Treatment for Hedychium villosum villosum

XML Treatment for Hedychium villosum tenuiflorum
